# Single-Cell Analysis Reveals Characterization of Infiltrating T Cells in Moderately Differentiated Colorectal Cancer

**DOI:** 10.3389/fimmu.2020.620196

**Published:** 2021-01-22

**Authors:** Xi Yang, Quan Qi, Yuefen Pan, Qing Zhou, Yinhang Wu, Jing Zhuang, Jiamin Xu, Mingyue Pan, Shuwen Han

**Affiliations:** ^1^Department of Oncology, Huzhou Cent Hospital, Affiliated Cent Hospital HuZhou University, Huzhou, China; ^2^Department of Critical Care Medicine, Huzhou Cent Hospital, Affiliated Cent Hospital HuZhou University, Huzhou, China; ^3^Graduate School of Second Clinical Medicine Faculty, Zhejiang Chinese Medical University, Hangzhou, China; ^4^Graduate School of Nursing, Huzhou University, Huzhou, China

**Keywords:** colon cancer, rectal cancer, single-cell RNA sequencing, tumor-infiltrating T cells, immunotherapy

## Abstract

**Objective:**

This study aimed to characterize the tumor-infiltrating T cells in moderately differentiated colorectal cancer.

**Methods:**

Using single-cell RNA sequencing data of isolated 1632 T cells from tumor tissue and 1252 T cells from the peripheral blood of CRC patients, unsupervised clustering analysis was performed to identify functionally distinct T cell populations, followed by correlations and ligand-receptor interactions across cell types. Finally, differential analysis of the tumor-infiltrating T cells between colon cancer and rectal cancer were carried out.

**Results:**

A total of eight distinct T cell populations were identified from tumor tissue. Tumor-Treg showed a strong correlation with Th17 cells. CD8^+^T_RM_ was positively correlated with CD8^+^IEL. Seven distinct T cell populations were identified from peripheral blood. There was a strong correlation between CD4+T_N_ and CD4+blood-T_CM_. Colon cancer and rectal cancer showed differences in the composition of tumor-infiltrating T cell populations. Tumor-infiltrating CD8^+^IEL cells were found in rectal cancer but not in colon cancer, while CD8^+^ T_N_ cells were found in the peripheral blood of colon cancer but not in that of rectal cancer. A larger number of tumor-infiltrating CD8^+^ Tex (88.94%) cells were found in the colon cancer than in the rectal cancer (11.06%). The T cells of the colon and rectal cancers showed changes in gene expression pattern.

**Conclusions:**

We characterized the T cell populations in the CRC tumor tissue and peripheral blood.

## Highlights

1. We profiled 1632 T cells from tumor tissue and 1252 T cells from peripheral blood of moderately differentiated CRC patients using scRNA-seq
2. T cells from the peripheral blood showed diverse phenotypes compared to those of T cells from the tumor tissue
3. A total of 7852 ligand-receptor pairs among eight T cell types were identified from the tumor tissue, and 4546 ligand-receptor pairs among seven T cell clusters were identified from the peripheral blood
4. Colon cancer and rectal cancer also showed differences in composition of tumor-infiltrating T cell populations
5. T cells from colon cancer and rectal cancer tissues showed changes in gene expression pattern

## Introduction

Colorectal cancer (CRC) is the fourth most fatal disease in the world with approximately 900 000 deaths per year (1). Colon and rectal cancers are considered as a single tumor entity, called CRC, because both develop in the large bowel, which is regarded as a single organ (2). However, there are differences in their etiology, histology, drug response, and prognosis, based on tumor location within the colon (approximately 150 cm) and rectum (approximately 16 cm) (3). Based on the CRC statistics of 2017, the proximal colon is the most common tumor location, accounting for 41% of CRC cases, while distal colon and rectum account for 21% and 25%, respectively, and the distribution of tumor location differs with age and gender (4). The risk of malignant transformation in the rectal mucosa is at least four times higher than that in the colon mucosa. In formal carcinogenesis, 85–90% of cancers occur mostly in the form of adenomas (5). Compared with that for colon cancer (total mesorectal excision), the surgery for rectal cancer presents more challenges and risks due to its anatomy and topographic position (6).

The immune system influences the development and progression of CRC, and the great advances in understanding tumor immune microenvironment provide novel therapeutic strategies for CRC (7). Some immune cell subsets have been regarded as promising targets for immunotherapy and in the study of clinical biomarkers, including T cells and natural killer (NK) cells (8, 9). Fujimoto et al. found that patients with adenocarcinoma showed significantly elevated CD4/CD8 ratio and regulatory T cells (Tregs), based on the paired analyses with normal mucosa, and the accumulation frequency of Tregs was related to the tumor’s local infiltration and extension (10). De Simone et al. reported that infiltrating Treg cells in tumors showed high suppressive activity, and up-regulation of several immune checkpoints and their ligands were found in tumor-infiltrating Treg cells, which provide potential targets in immunotherapy (11). The tumor-infiltrating T lymphocytes (TILs) are essential for effective anti-tumor immune response, and high level of TILs has been reported to be related to better prognostic survival in CRC (12–14).

Intratumoral heterogeneity (phenotypic diversity, such as (epi)genetic abnormality and cell surface markers) continues to be a challenge in cancer treatment (15). The developed single-cell RNA sequencing (scRNA-seq) technologies recently showed incomparable superiority in evaluating tumor cellular heterogeneity (16). Based on the immune atlas constructed by scRNA-seq of isolated immune cells from tissue (normal and breast cancer), peripheral blood, and lymph node, Azizi et al. found that there was enormous diversity in immune cells. They suggested that T cells and myeloid cells were the two major cellular targets in tumor immunotherapy, and the T cells in peripheral blood and lymph node showed diverse phenotypes in comparison with that of tumor tissue (17). Using scRNA-seq, Chung et al. characterized the heterogeneous tumor cells and the surrounding immune and stromal cells, and they found that the majority of non-cancer cells were mainly macrophages, T and B lymphocytes, in which both macrophages (M2 phenotype) and lymphocytes (regulatory/exhausted phenotype) were immunosuppressive phenotypes (18).

Based on scRNA-seq of T cells and T cell receptor tracking, Zhang et al. identified 20 T cell subsets in CRC and comprehensively dissected the T cell properties (functions and clonalities), uncovering the development and migration of T cells within tumors (19). Azizi et al. had reported that T cells in the peripheral blood and lymph node showed diverse phenotypes compared to those of the T cells in the breast cancer tissue (17). Therefore, using the scRNA-seq data of Zhang et al., we further investigated the characteristics of isolated T cells from the tumor tissue and peripheral blood in this study. In the study by Zhang et al., the 12 CRC patients showed different pathological types (one patient was well-differentiated, five patients were moderately differentiated, two patients were poorly or moderately differentiated, and four patients were poorly differentiated), which could have been a confounding factor for the results. Therefore, only the moderately differentiated samples, accounting for the maximum proportion, were selected and used in our analysis. In addition, considering the similarities and differences between colon and rectal cancers, the differences in tumor-infiltrating T cells and peripheral blood T cells between colon cancer and rectal cancer, which have not yet been reported in CRC, were determined in this study. The findings of this study provide novel insights and potential targets in the immunotherapy of CRC.

## Materials and Methods

### Data Acquisition

Following the method of Zhang et al. (19), the normalized gene expression matrix files for each cell were acquired. A total of 12 patients with rectum adenocarcinoma or colon adenocarcinoma (one well differentiated, five moderately differentiated, two poorly or moderately differentiated, and four poorly differentiated) were included in the data set, of which five patients (moderately differentiated) were enrolled in our current study (**Table S1**). The original data set contained scRNA-seq data of different subtypes of T cells collected from tumor tissue, adjacent normal mucosa, and peripheral blood of the patients. The data from the tumor tissue and peripheral blood were used in the current analysis. After data screening, the gene expression matrix files for each tumor tissue cell of the five moderately differentiated patients were obtained, including 1632 cells and 12,547 genes. Similarly, the gene expression matrix files for each cell from the peripheral blood of the five moderately differentiated patients were obtained, including 1,252 cells and 12,547 genes. All the analyses on the tumor tissue and peripheral blood data were performed independently.

### Unsupervised Cluster Analysis and Cell Type Identification

The cells were divided into two major categories, based on previous flow cell sorting, including CD8+ T cells and CD4+ T cells. To reach a well-clustered result, clustering analysis was firstly performed for CD8+ T cells and CD4+ T cells and then the obtained cell clusters were merged. In brief, the top 1500 highly variable genes were selected using the sscClust package (20) (https://github.com/Japrin/sscClust), followed by the analysis of dimension reduction based on the Spearman correlation coefficient between cells. K-means was used for the clustering analysis. A total of 2–20 cell clusters were pre-set. The normalized mutual information (NMI) index was selected to determine the number of final cell clusters, and a larger NMI index represents a more accurate clustering result. The clustering results were visualized using t-distributed stochastic neighbor embedding (tSNE). The marker genes of each cell cluster in the study of Zhang et al. were used to define the obtained cell clusters. The heatmap and violin plot for each gene across cell clusters showed their expression in different cell clusters, and the cell cluster with significantly high expression of marker genes was defined as the cell type that corresponded with the marker genes.

### Identification of Correlation and Cell-Cell Crosstalk Between Cell Clusters

Based on the average expression of each gene in cell clusters, the Pearson correlation coefficient between cell clusters was calculated using the Cor function of the R language, and the Corrplot (21) (https://mirrors.tuna.tsinghua.edu.cn/CRAN/web/packages/corrplot) of the R package was used to plot the correlation heatmap. The iTALK (22) (https://github.com/Coolgenome/iTALK) of the R package was used to investigate the cell-cell crosstalk between cell clusters. Briefly, the top 50% highly expressed genes of each cell cluster were selected and matched to the 2,648 non-redundant ligand-receptor pairs included in the iTALK package. There are four categories of ligand-receptor pairs, according to the ligand types, including growth factor, cytokine, checkpoint, and other ligand-receptor pairs. The top 20 ligand-receptor pairs for each type were presented by the ligand-receptor interaction network.

### Differential Expression Analysis for Infiltrating T Cells Between Colon Cancer and Rectal Cancer

To analyze the differences between colon cancer and rectal cancer at the cellular level, the differential analysis of colon cancer vs. rectal cancer was performed using the classical Bayesian method of the Limma package (23). For each cell cluster, the differentially expressed genes (DEGs) in colon cancer vs. those in rectal cancer were screened with an adjusted *P* value < 0.05 and |logFC|>1 after multiple tests correction, using the Benjamini & Hochberg method.

### Enrichment Analysis and Protein-Protein Interactions for DEGs

For the DEGs in each cell cluster, enrichment analysis was performed using the online tool, Metascape (24) (http://metascape.org) to investigate the involved functional terms, including biological process terms in gene ontology, KEGG pathways, and Reactome pathways. The parameters were set as: Min Overlap = 3, *P* value cutoff = 0.05, and Min Enrichment = 1.5. The protein-protein interactions (PPI) for DEGs were retrieved from the BioGrid (25), InWeb_IM (26), and OmniPath (27) databases using the online tool Metascape, with default parameters (Min Network Size=3 and Max Network Size=500). Based on the obtained interactions, the PPI network was visualized using Cytoscape software (28) (version 3.4.0, http://chianti.ucsd.edu/cytoscape-3.4.0/). In addition, the Molecular Complex Detection (MCODE) algorithm (29) of Metascape was used to identify the modules of the PPI network. Enrichment analysis was also performed for the genes in modules.

## Results

### Part 1: Analysis Results for Data From the Tumor Tissue

#### Identification of Different Tumor-Infiltrating T Cell Types

Unsupervised clustering analysis using K-means revealed that the CD4+ T cells were clustered into 13 clusters based on the maximal NMI index (**Figure S1A**). After merging similar cell clusters, four clusters of CD4+ T cells were identified—CD4_C05-CXCR6, CD4_C07-GZMK, CD4_C08-IL23R, and CD4_C12-CTLA4 (**Table S2A**). Similarly, CD8+ T cells were grouped into 12 clusters based on the maximal NMI index (**Figure S1B**). After merging similar cell clusters, four clusters of CD8+ T cells were identified—CD8_C04-GZMK, CD8_C05-CD6, CD8_C06-CD160, and CD8_C07-LAYN (**Table S2B**).

**Figure 1:**
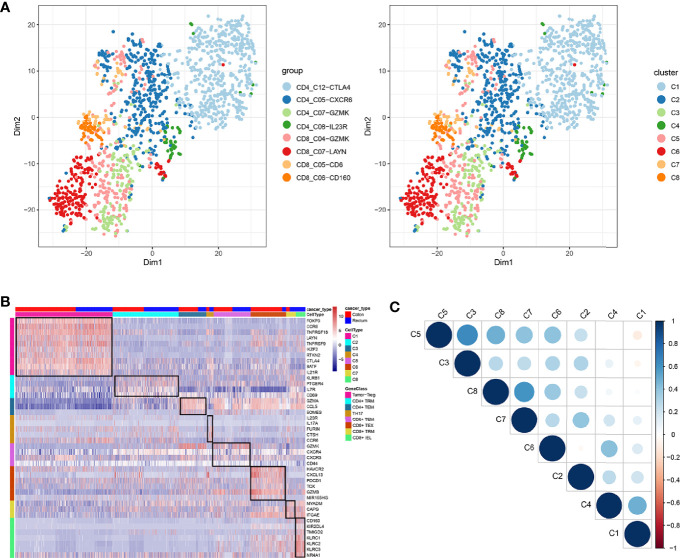
Identification of distinct T cell types from CRC tumor tissue. **(A)** tSNE analysis of T cells shows eight distinct clusters of T cells. Different colors represent different cell clusters; **(B)** Heatmap of marker genes across eight T cells clusters. The red clocks and blue blocks in upper strata represent T cells from colon cancer and rectal cancer, respectively; marker genes are shown in rows; the colored blocks in the left side and top represent the eight T cell clusters; **(C)** Correlations across the eight T cell clusters. Node size represents the absolute value of the correlation coefficient; blue and red nodes represent positive correlations and negative correlations.

**Figure 2:**
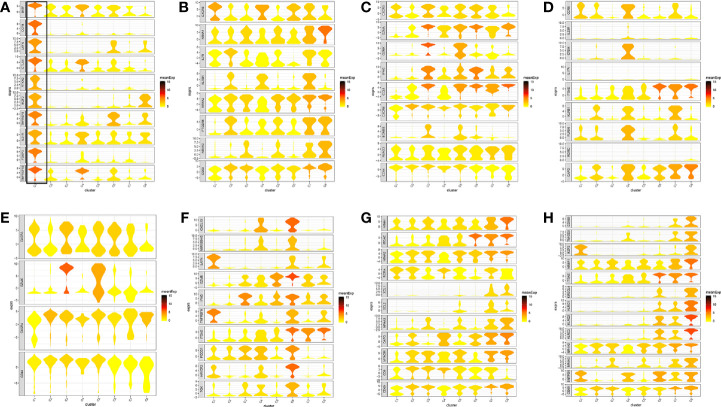
Marker genes specifically expressed in the eight cell clusters (tumor tissue). The violin plot for each gene shows the distribution and relative expression for marker genes of the eight T cell clusters. **(A)** tumor-Treg; **(B)** CD4+ tissue-resident memory T cells; **(C)** CD4+ effector memory T cells; **(D)** T help 17 cell; **(E)** CD8+ effector memory T cells; **(F)** CD8+ exhausted T cell; **(G)** CD8+ tissue-resident memory T cells; **(H)** CD8+ intraepithelial lymphocyte. For panel **(A)** in Figure 2, the gene on the left represents the marker genes of tumor-Treg. Horizontal axis represents the 8 identified cell clusters (C1 to C8). It could be seen that the marker genes of tumor-Treg were significant highly expressed in C1. Therefore, C1 cells were defined as tumor-Treg.

In total, eight cell clusters were identified (**Figure 1A**). The marker genes of the eight cell clusters were used to further validate the defined cell clusters (**Figures 1B** and **2**). High expression of CD4_C12-CTLA4 (tumor regulatory T cell, Tumor-Treg) markers, such as CCR8, RTKN2, and FOXP3, was observed in C1. High expression of CD8_C07-LAYN (CD8^+^ intraepithelial lymphocyte, CD8^+^IEL) markers, such as CD160, KLRC3, and KLRC2, was observed in C8, suggesting that the definitions of cell clusters were accurate. The specific functional annotations of cell clusters were shown in http://crc.cancer-pku.cn/. Among the eight cell clusters, there was a strong correlation between C1 (Tumor-Treg) and C4 (T help 17 cell, Th17), while C1 and C4 showed a weak correlation with other cell clusters. C5 (CD8^+^ effector memory T cell, CD8^+^T_EM_) showed a strong correlation with different cell clusters, including C3 (CD4^+^ effector memory T cell, CD4^+^T_EM_), C8 (CD8^+^IEL), C7 (CD8^+^ Tissue-resident memory T cell, CD8^+^ T_RM_), and C6 (CD8^+^ exhausted T cell, CD8^+^ T_EX_) (**Figure 1C**).

#### The Ligand-Receptor Pairs in Tumor-Infiltrating T Cells Crosstalk

**Figure 3:**
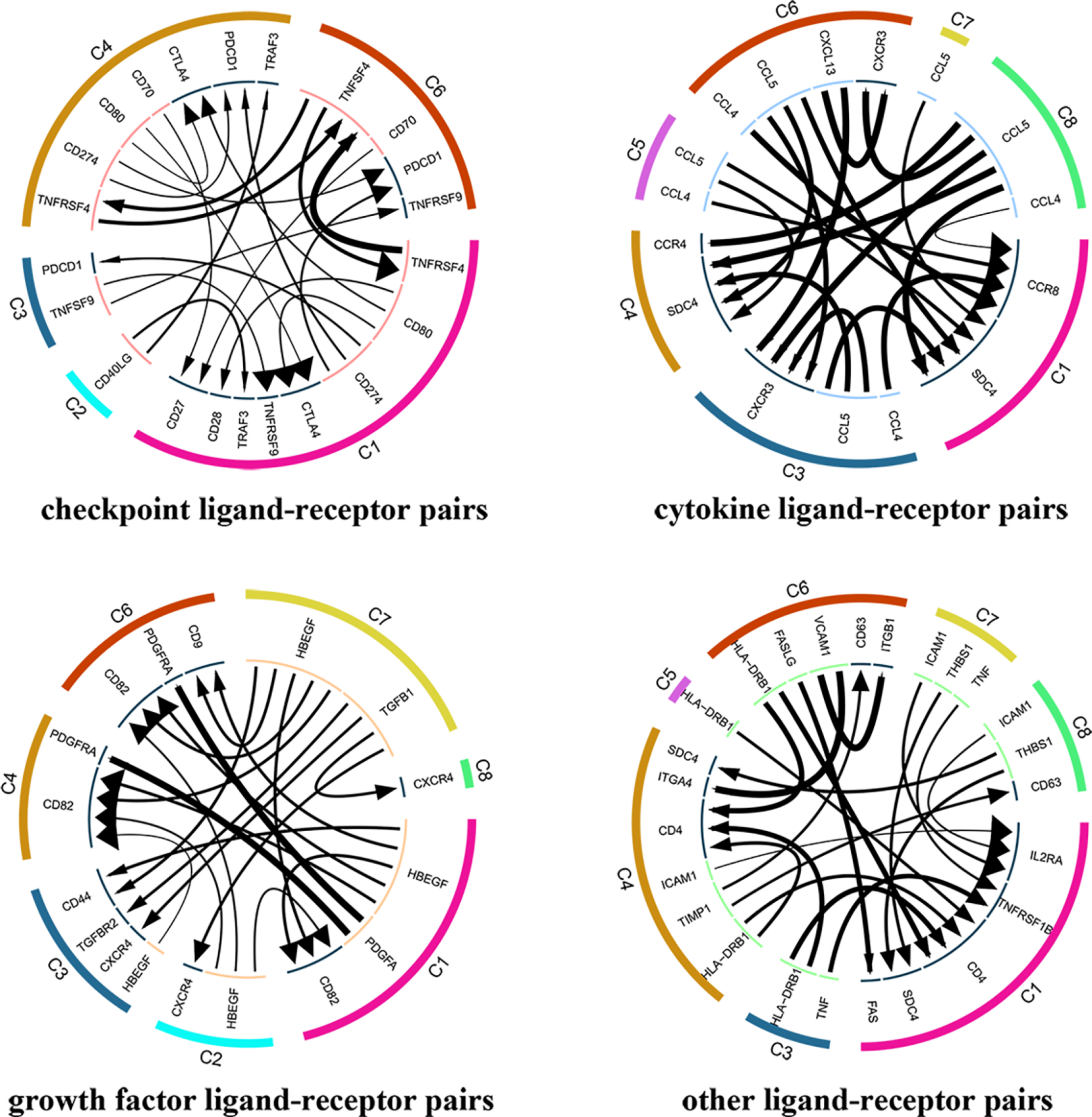
Analysis of cellular crosstalk *via* ligand-receptor interactions (tumor tissue). Cyclic graphs show the top 20 ligand-receptor interactions for growth factor type, cytokine type, checkpoint type, and other types. Colors in the outer circle represent different cell clusters; Colors in the inner circle represent different gene types; arrows point to the receptor, and larger arrows represent higher expression level.

A total of 7852 ligand-receptor pairs were screened among the eight cell clusters, including 636 growth factors, 1170 cytokines, 395 checkpoints, and 5651 other ligand-receptor pairs. The top 20 ligand-receptor pairs for each type were presented by the ligand-receptor interaction network (**Figure 3**). C1 (Tumor-Treg), C4 (Th17), and C6 (CD8^+^ T_EX_) showed more crosstalk with other cell clusters. For example, a cytokine ligand-receptor pair (CCL4-CCR8) was found between C6 (CD8^+^ T_EX_) and C1 (Tumor-Treg). Checkpoint ligand-receptor pairs (e.g., tumor Treg CD80-Th17 cell CTLA4 and tumor Treg CD274-Th17 cell PDCD1) were identified.

#### Differences in Tumor-Infiltrating T Cells Between Colon Cancer and Rectal Cancer

**Figure 4:**
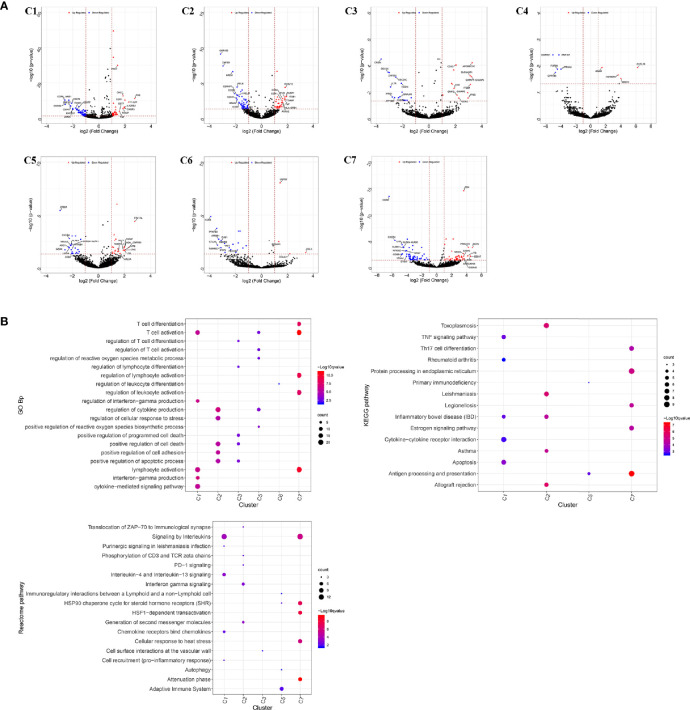
Differentially expressed genes in tumor-infiltrating T cells between colon cancer and rectal cancer. **(A)** Volcano plot shows the up-regulated and down-regulated genes in the T cell clusters between colon cancer and rectal cancer; **(B)** Enrichment analysis for the differentially expressed genes in tumor-infiltrating T cells between colon cancer and rectal cancer. Bubble diagram shows the top 5 gene ontology biological processes, KEGG pathways, and Reactome pathways for differentially expressed genes in tumor-infiltrating T cells between colon cancer and rectal cancer. Vertical axis shows terms of biological processes and pathways, and horizontal axis represents the genes in different cell clusters. Larger node size represents the larger ratio of enriched genes/total genes. The color from blue to red represents P value from large to small. C1, tumor-Treg; C2, CD4+ tissue-resident memory T cells; C3, CD4+ effector memory T cells; C5, CD8+ effector memory T cells; C6, CD8+ exhausted T cell; C7, CD8+ tissue-resident memory T cells. For C8 (CD8+ intraepithelial lymphocyte), differential analysis was not performed for that all the cells were from rectal cancer.

The infiltrating T cells in colon cancer and rectal cancer were counted (**Table 1A**). It could be seen that C1 included 547 infiltrating Treg cells, of which 339 were in colon cancer, and 208 were in rectal cancer. C6 included 199 infiltrating CD8^+^ T_EX_ cells, and the majority of the cells were in colon cancer (177 cells, 88.94%) compared to those in rectal cancer (22 cells, 11.06%). C8 included 54 infiltrating CD8^+^IEL cells, which were all in rectal cancer. Therefore, C8 was excluded from the differential expression analysis. The screened DEGs are shown in **Table 1B**. C1 (Tumor-Treg) showed 112 DEGs between colon cancer and rectal cancer, such as TNF and CXCR3, which were up-regulated, while CXCR6 and CCR6 were down-regulated in colon cancer in comparison to that of rectal cancer. For C4 (Th17), only nine DEGs, including TNFRSF9 and CXCL13 were screened between colon cancer and rectal cancer (**Figure 4A** and **Figure S2**).

**Table 1:** Differences for tumor infiltrating T cells numbers and DEGs between colon cancer and rectal cancer.

(A) Number of Cells			(B) Number of DEGs			
Clusters	Colon cancer	Rectum cancer	Clusters	Up-regulated	Down-regulated	Total
C1	339	208	C1	43	69	112
C2	175	197	C2	37	55	92
C3	109	46	C3	14	16	30
C4	17	25	C4	4	5	9
C5	105	103	C5	23	23	46
C6	177	22	C6	4	18	22
C7	21	34	C7	51	54	105
C8	0	54				

**Figure 5:**
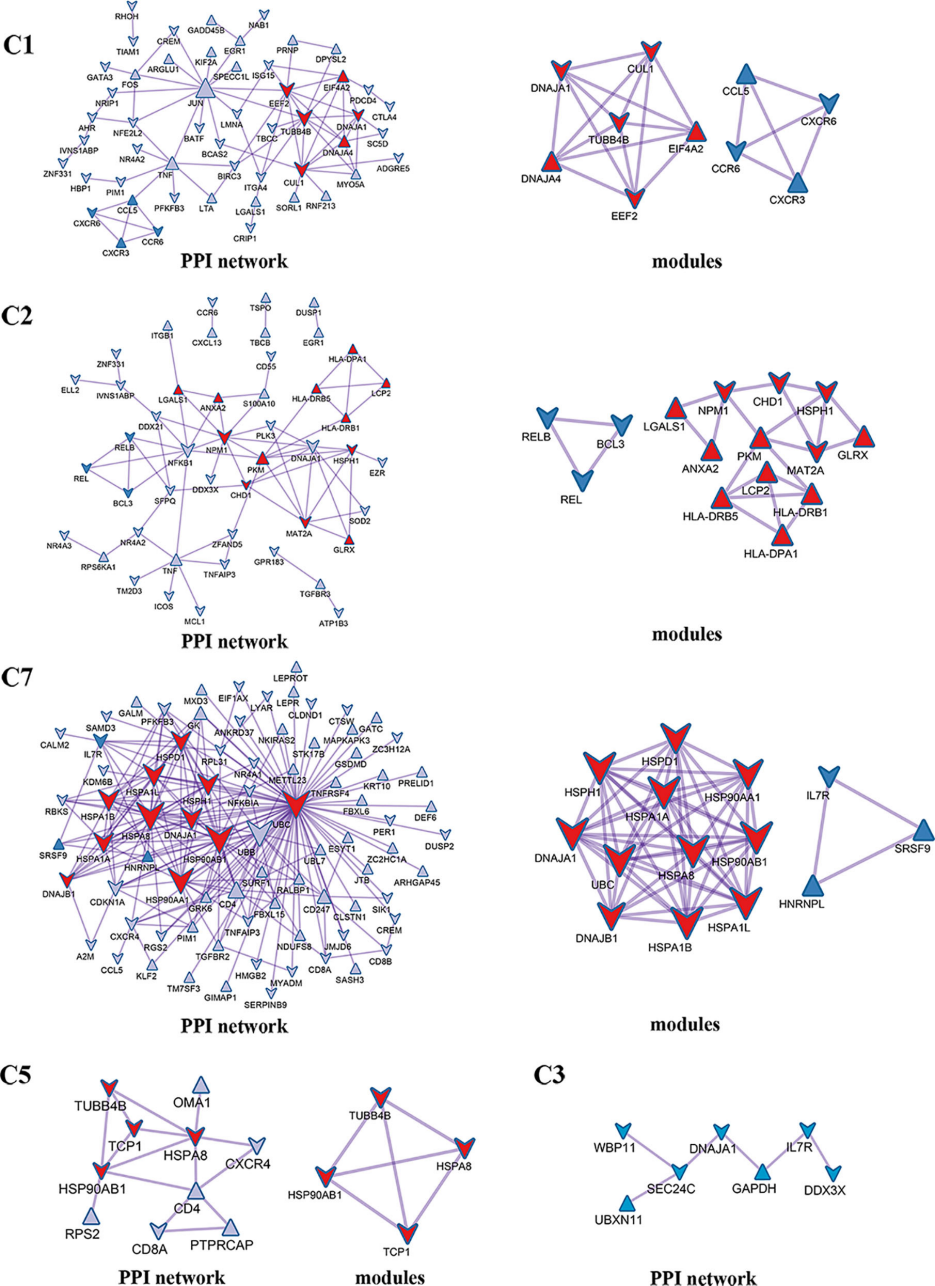
PPI network and modules for differentially expressed genes in tumor-infiltrating T cells between colon cancer and rectal cancer. PPI network shows the interactions for differentially expressed genes in tumor-infiltrating T cells between colon cancer and rectal cancer. Triangle nodes represent the up-regulated genes and V-shaped nodes represent the down-regulated genes; node size represent the degree of the node in network; red nodes represent genes in module 1 and blue nodes represent genes in module 2. C1, tumor-Treg; C2, CD4+ tissue-resident memory T cells; C3, CD4+ effector memory T cells; C5, CD8+ effector memory T cells; C7, CD8+ tissue-resident memory T cells.

Enrichment analysis showed that there were similarities and differences between the enriched functions and pathways for those DEGs (**Figure 4B**). For example, the DEGs in C7 (CD8^+^ T_RM_) were enriched in T cell differentiation, T cell activation, lymphocyte activation, and regulation of lymphocyte activation, of which T cell activation and lymphocyte activation were similar to that of C1 (Tumor-Treg). We further investigated the interactions between those DEGs and constructed the PPI network (**Figure 5**). Besides, the modules were identified from the PPI network, and enrichment analysis was also performed for the genes in modules (**Table 2**). For example, for the DEGs in C1 (Tumor-Treg), the PPI network contained 51 genes and 80 interactions, and two modules were identified from the PPI network. The genes in module 1, including *DNAJA1*, *EEF2*, and *TUBB4B*, were enriched in cellular responses to stress and cellular responses to external stimuli pathways, while the genes in module 2 (*CCL5*, *CCR6*, *CXCR3*, and *CXCR6*) were implicated in pathways, including chemokine receptors bind chemokines, chemokine-mediated signaling pathway, and cellular response to chemokines.

**Table 2:** The top 5 significantly enriched function terms and pathways for genes in modules (Analysis results for data from tumor tissue).

Name	Category	Description	Count	Log(q-value)
C1_MCODE_1	Reactome Gene Sets	HSP90 chaperone cycle for steroid hormone receptors (SHR)	3	-5.1
C1_MCODE_1	Reactome Gene Sets	Cellular responses to stress	3	-2.7
C1_MCODE_1	Reactome Gene Sets	Cellular responses to external stimuli	3	-2.6
C1_MCODE_2	Reactome Gene Sets	Chemokine receptors bind chemokines	4	-8.4
C1_MCODE_2	GO Biological Processes	Chemokine-mediated signaling pathway	4	-7.8
C1_MCODE_2	GO Biological Processes	Cellular response to chemokine	4	-7.6
C1_MCODE_2	GO Biological Processes	Response to chemokine	4	-7.6
C1_MCODE_2	KEGG Pathway	Chemokine signaling pathway	4	-6.7
C2_MCODE_1	Reactome Gene Sets	Generation of second messenger molecules	12	-6.9
C2_MCODE_1	Reactome Gene Sets	Translocation of ZAP-70 to Immunological synapse	12	-5.4
C2_MCODE_1	Reactome Gene Sets	Phosphorylation of CD3 and TCR zeta chains	12	-5.3
C2_MCODE_1	Reactome Gene Sets	PD-1 signaling	12	-5.2
C2_MCODE_1	Reactome Gene Sets	TCR signaling	12	-5
C2_MCODE_2	GO Biological Processes	NIK/NF-kappaB signaling	3	-4.9
C2_MCODE_2	GO Biological Processes	I-kappaB kinase/NF-kappaB signaling	3	-4.5
C2_MCODE_2	GO Biological Processes	Negative regulation of cytokine production	3	-4.3
C2_MCODE_2	GO Biological Processes	Regulation of cytokine production	3	-3.4
C5_MCODE_1	Reactome Gene Sets	HSP90 chaperone cycle for steroid hormone receptors (SHR)	3	-5.7
C5_MCODE_1	Reactome Gene Sets	Autophagy	3	-4.6
C5_MCODE_1	GO Biological Processes	Protein folding	3	-4.2
C5_MCODE_1	GO Biological Processes	Regulation of protein stability	3	-3.9
C5_MCODE_1	Reactome Gene Sets	Neutrophil degranulation	3	-3.4
C7_MCODE_1	Reactome Gene Sets	Attenuation phase	7	-17
C7_MCODE_1	GO Biological Processes	Response to heat	10	-17
C7_MCODE_1	GO Biological Processes	Response to unfolded protein	10	-17
C7_MCODE_1	GO Biological Processes	Response to topologically incorrect protein	10	-16
C7_MCODE_1	GO Biological Processes	Protein folding	10	-16

### Part 2: Analysis Results for Data From the Peripheral Blood

#### Identification of Different Infiltrating T Cell Types

Unsupervised clustering analysis using K-means revealed that the CD4^+^ T cells were clustered into 13 clusters based on the maximal NMI index (**Figure S3A**). After merging similar cell clusters, four cell clusters of CD4^+^ T cells were identified—CD4_C01-CCR7, CD4_C02-ANXA1, CD4_C03-GNLY, and CD4_C10-FOXP3 (**Table S3A**). Similarly, CD8+ T cells were grouped into seven clusters based on the maximal NMI index (**Figure S3B**). After merging similar cell clusters, four cell clusters of CD8^+^ T cells were identified—CD8_C01-LEF1, CD8_C02-GPR183, and CD8_C03-CX3CR1 (**Table S3B**).

**Figure 6:**
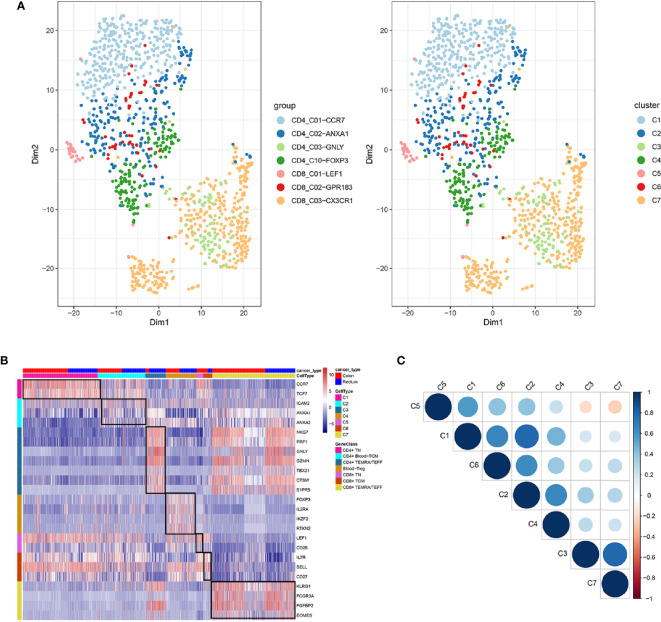
Identification of distinct T cell types from CRC peripheral blood. **(A)** tSNE analysis of T cells shows eight distinct clusters of T cells. Different colors represent different cell clusters; **(B)** Heatmap of marker genes across eight T cells clusters. The red clocks and blue blocks in upper strata represent T cells from color cancer and rectal cancer; marker genes are shown in rows; the colored blocks in left side and top represent the eight T cell clusters; **(C)** correlations across the eight T cell clusters. Node size represents the absolute value of the correlation coefficient; blue and red nodes represent positive correlations and negative correlations.

**Figure 7:**
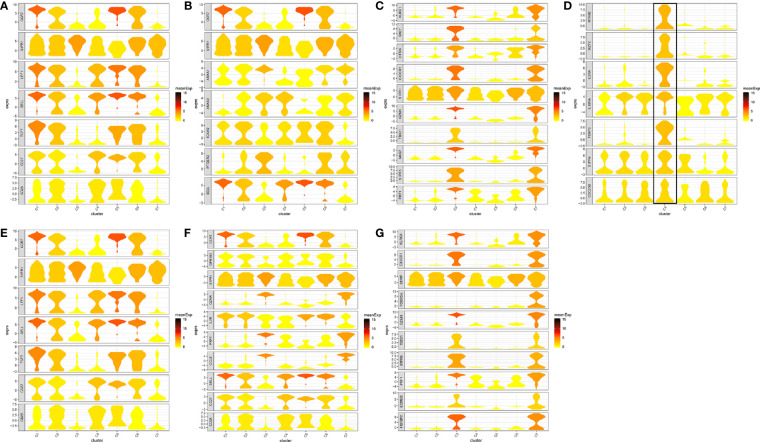
Marker genes specifically expressed in the seven cell clusters (peripheral blood). The violin plot for each gene shows the distribution and relative expression for marker genes of the seven T cell clusters. **(A)** CD4+ Naive T cell. **(B)** CD4+ central memory T cell; **(C)** CD4+ recently activated effector memory T cells; **(D)** blood-Treg; **(E)** CD8+ Naive T cell; **(F)** CD8+ central memory T cell; **(G)** CD8+ recently activated effector memory T cells. For the panel **(D)** in **Figure 7**, the gene on the left represents the marker genes of blood-Treg. Horizontal axis represents the seven identified cell clusters (C1 to C8). It could be seen that the marker genes of blood-Treg were significantly highly expressed in C4. Therefore, C4 was defined as tumor-Treg.

In total, seven cell clusters were identified (**Figure 6A**). The marker genes of the seven cell clusters were used to further validate the defined cell clusters (**Figures 6B** and **7**). High expression of CD4_C10-FOXP3 (Blood regulatory T cell, Blood-Treg) markers, such as RTKN2, IKZF2, and FOXP3, were observed in C4, suggesting that the definition of cell clusters was accurate. The specific functional annotations of cell clusters were shown in http://crc.cancer-pku.cn/. C1 was the cluster of CD4^+^ Naive T cells (CD4^+^ T_N_), C2 was the cluster of CD4^+^ central memory T cells (CD4^+^ blood-T_CM_), C3 was the cluster of CD4^+^ recently activated effector memory T cells (CD4^+^ T_EMRA_/T_EFF_), C5 was the cluster of CD8^+^ T_N_ cells, C6 was the cluster of CD8^+^T_CM_ cells, and C7 was the cluster of CD8^+^ T_EMRA_/T_EFF_ cells.

Among the seven cell clusters, there was a strong correlation between C3 (CD4^+^ T_EMRA_/T_EFF_) and C7 (CD8^+^ T_EMRA_/T_EFF_), while C3 and C7 showed a weak correlation with the other cell clusters. Besides, C1(CD4^+^ T_N_) and C2 (CD4^+^ blood-T_CM_) showed a strong correlation (**Figure 6C**).

#### The Ligand-Receptor Pairs in Blood T Cells Crosstalk

**Figure 8:**
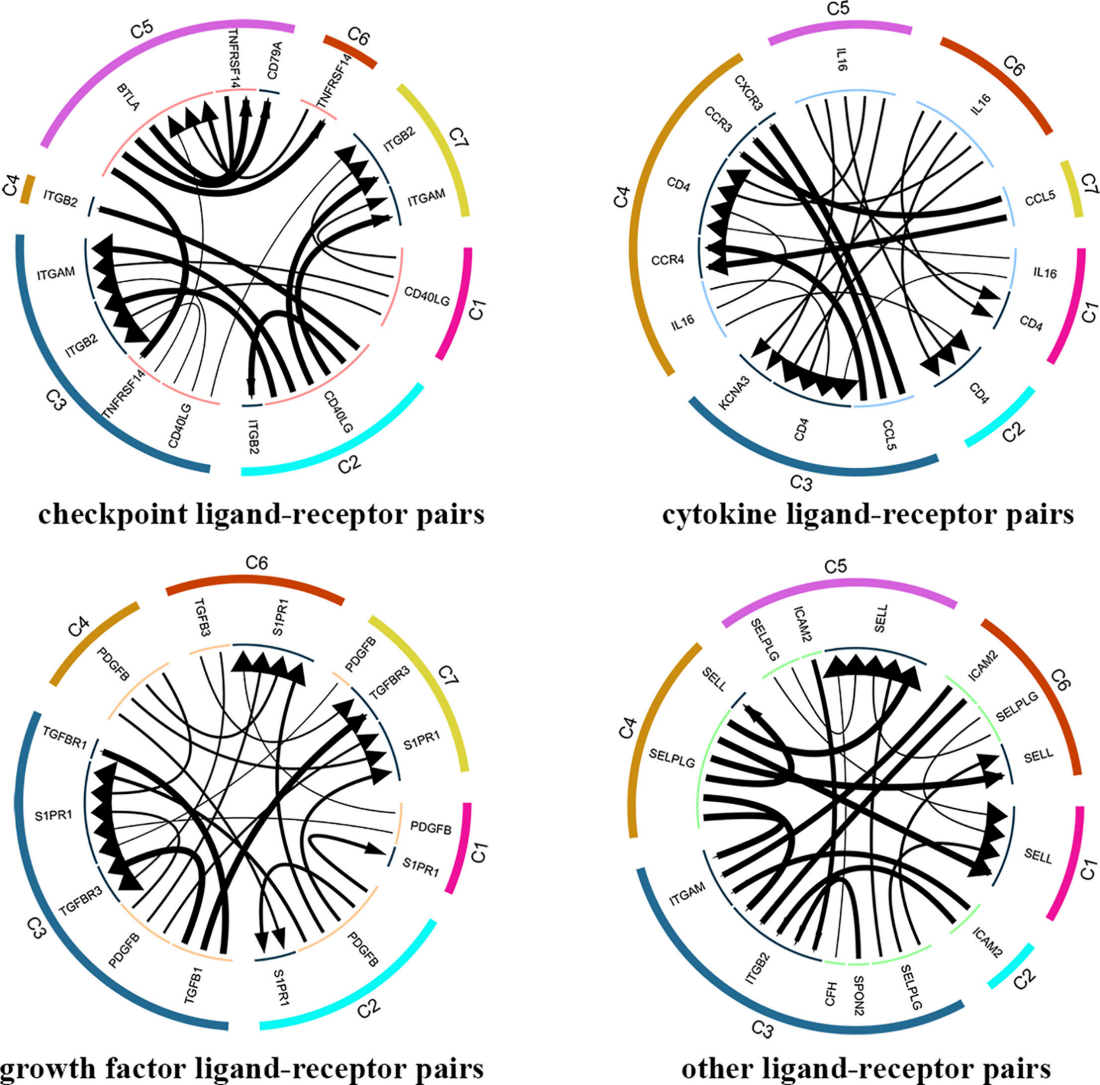
Analysis of cellular crosstalk *via* ligand-receptor interactions (peripheral blood). Cyclic graphs show the top 20 ligand-receptor interactions for growth factor type, cytokine type, checkpoint type, and other types. Colors in outer circle represent different cell clusters; Colors in inner circle represents different gene types; arrows point to the receptor, and larger arrow represent higher expression level.

In total, 4546 ligand-receptor pairs were screened among the seven cell clusters, including 263 growth factors, 448 cytokines, 168 checkpoints, and 3,667 other ligand-receptor pairs. The top 20 ligand-receptor pairs for each type were presented by the ligand-receptor interaction network (**Figure 8**). C3 (CD4^+^ T_EMRA_/T_EFF_) showed more crosstalk with other cell clusters. For example, a cytokine ligand-receptor pair (CCL5-CXCR3/CCR3) was found between C3 (CD4^+^ T_EMRA_/T_EFF_) and C4 (Blood-Treg), and other ligand-receptor pair (CFH-SELL) was found between C3 (CD4^+^ T_EMRA_/T_EFF_) and C5 (CD8^+^ T_N_).

#### Differences for Blood T Cells Between Colon Cancer and Rectal Cancer

**Figure 9:**
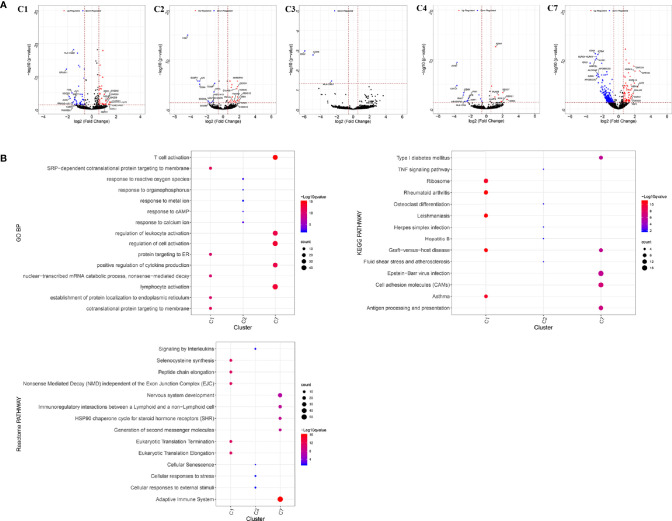
Differentially expressed genes in blood T cells between colon cancer and rectal cancer. **(A)** Volcano plot shows the up-regulated and down-regulated genes in the T cell clusters between colon cancer and rectal cancer; **(B)** Enrichment analysis for the differentially expressed genes in blood T cells between colon cancer and rectal cancer. Bubble diagram shows the top 5 Gene Ontology biological processes, KEGG pathways and Reactome pathways for differentially expressed genes in the peripheral blood T cells between colon cancer and rectal cancer. Vertical axis shows terms of biological processes and pathways, and horizontal axis represents the genes in different cell clusters. Larger node size represents the larger ratio of enriched genes/total genes. The color from blue to red represents P value from large to small. C1, CD4+ Naive T cell. C2, CD4+ central memory T cell; C3, CD4+ recently activated effector memory T cells; C4, blood-Treg; C7, CD8+ recently activated effector memory T cells.

The number of blood T cells in colon cancer and rectal cancer were counted (**Table 3A**). It could be seen that C1 included 344 blood CD4^+^ Naive T cells, of which 208 were in colon cancer, and 136 were in rectal cancer. C4 included 142 Blood-Treg cells, of which colon cancer (65 cells) and rectal cancer (77 cells) shared a similar number of Blood-Treg cells. C5 included 29 CD8^+^ Naive T cells, which were all in colon cancer. Therefore, C5 was excluded from the differential expression analysis. The screened DEGs are shown in **Table 3B**. For C7 (CD8^+^ T_EMRA_/T_EFF_), there were 359 DEGs between colon cancer and rectal cancer, such as HSPA1L and IL7R were up-regulated, while CD8A and CTSW were down-regulated in colon cancer compared to that in rectal cancer. For C3 (CD4^+^ T_EMRA_/T_EFF_), only three DEGs were screened between colon cancer and rectal cancer, and no DEGs were found for C6 (CD8^+^T_CM_) (**Figure 9A** and **Figure S4**).

**Table 3:** Differences for blood T cells numbers and DEGs between colon cancer and rectal cancer.

(A) Number of cells			(B) Number of DEGs			
Clusters	Colon cancer	Rectum cancer	Clusters	Up-regulated	Down-regulated	Total
C1	208	136	C1	40	42	82
C2	111	108	C2	13	10	23
C3	17	77	C3	0	3	3
C4	65	77	C4	8	12	20
C5	29	0	C6	0	0	0
C6	22	21	C7	69	290	359
C7	244	137				

**Figure 10:**
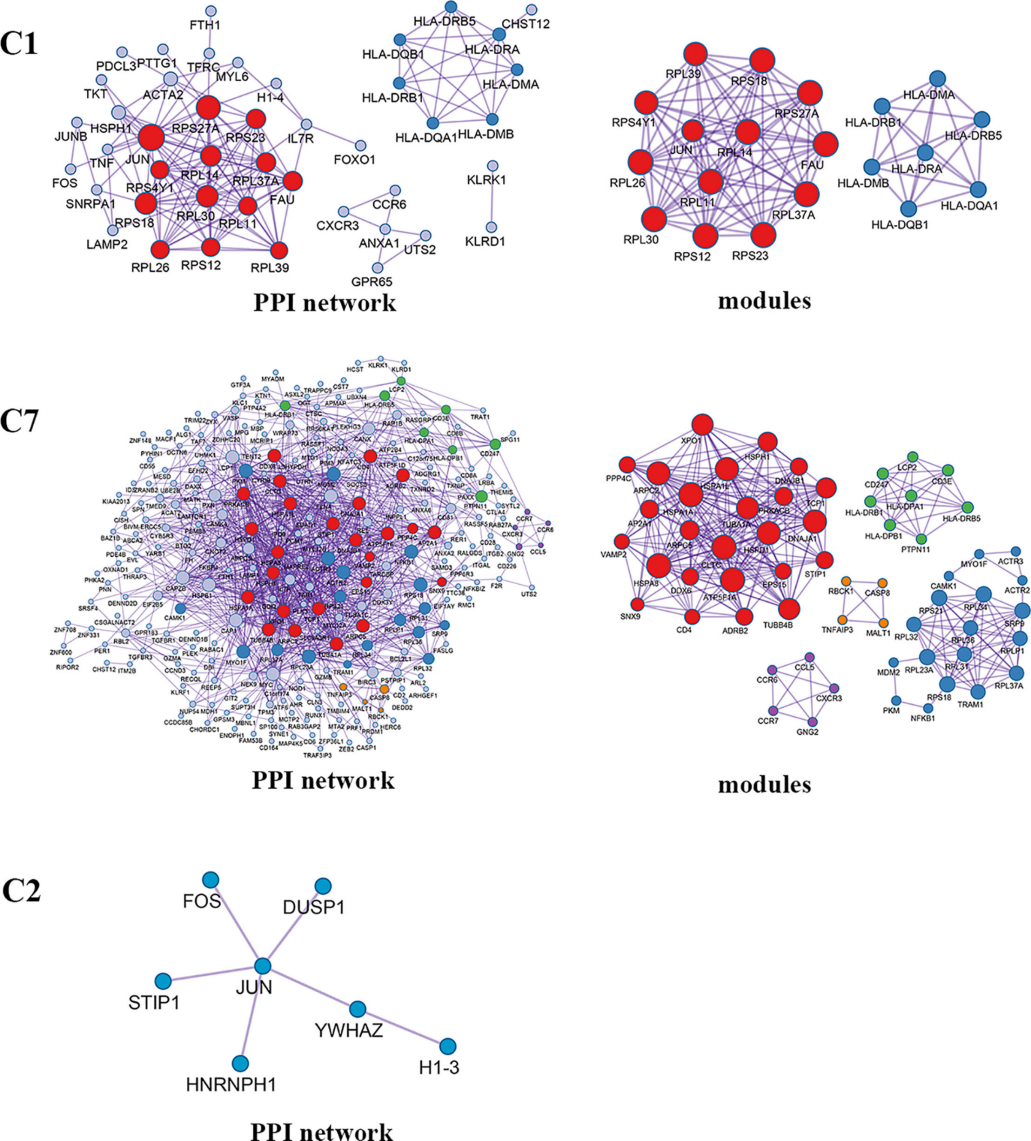
PPI network and modules for differentially expressed genes in blood T cells between colon cancer and rectal cancer. PPI network shows the interactions for differentially expressed genes in blood T cells between colon cancer and rectal cancer. Triangle nodes represent the up-regulated genes and V-shaped nodes represent the down-regulated genes; node size represent the degree of the node in network; different color represent different modules; C1, CD4+ Naive T cell. C2, CD4+ central memory T cell; C7, CD8+ recently activated effector memory T cells.

Enrichment analysis showed that the DEGs in different cell clusters were enriched in different functions and pathways (**Figure 9B**). For example, DEGs in C7 (CD8^+^ T_EMRA_/T_EFF_) were enriched in the adaptive immune system, antigen processing and presentation pathways, as well as in lymphocyte activation, T cell activation, and other biological processes. The interactions between those DEGs were further investigated to construct the PPI network and its modules (**Figure 10**). In addition, enrichment analysis was also performed for the genes in modules (**Table 4**). For example, for the DEGs in C7 (CD8^+^ T_EMRA_/T_EFF_), the PPI network contained 265 genes and 1,205 interactions, and five modules were identified from the PPI network. The genes in different modules were enriched in different biological processes and pathways. The genes in module 4 included chemokine *CCL5* and chemokine receptors *CCR6*, *CCR7*, and *CXCR3*, and were enriched in the chemokine signaling pathway, in response to chemokines. The genes in module 5 (*TNFAIP3*, *MALT1*, *CASP8*, and *RBCK1*) were implicated in signaling, including TNF signaling and I-kappaB kinase/NF-kappaB signaling, while the genes in module 3, including *HLA-DRB1*, *HLA-DPB1*, and *CD247*, were involved in PD-1 signaling, translocation of ZAP-70 to immunological synapse, and costimulation by the CD28 family.

**Table 4:** The top 5 significantly enriched function terms and pathways for genes in modules (analysis results for data from peripheral blood).

Name	Category	Description	Count	Log(q-value)
C1_MCODE_1	KEGG Pathway	Ribosome	12	-23
C1_MCODE_1	Reactome Gene Sets	Peptide chain elongation	11	-23
C1_MCODE_1	Reactome Gene Sets	Eukaryotic Translation Elongation	11	-23
C1_MCODE_1	Reactome Gene Sets	Selenocysteine synthesis	11	-23
C1_MCODE_1	Reactome Gene Sets	Eukaryotic Translation Termination	11	-23
C1_MCODE_2	KEGG Pathway	Asthma	7	-19
C1_MCODE_2	KEGG Pathway	Allograft rejection	7	-18
C1_MCODE_2	KEGG Pathway	Graft-versus-host disease	7	-18
C1_MCODE_2	KEGG Pathway	Type I diabetes mellitus	7	-18
C1_MCODE_2	KEGG Pathway	Intestinal immune network for IgA production	7	-17
C7_MCODE_1	Reactome Gene Sets	Clathrin-mediated endocytosis	10	-14
C7_MCODE_1	Reactome Gene Sets	HSP90 chaperone cycle for steroid hormone receptors (SHR)	8	-14
C7_MCODE_1	Reactome Gene Sets	Vesicle-mediated transport	13	-12
C7_MCODE_1	Reactome Gene Sets	Membrane Trafficking	12	-11
C7_MCODE_1	GO Biological Processes	’De novo’ protein folding	6	-10
C7_MCODE_2	GO Biological Processes	SRP-dependent cotranslational protein targeting to membrane	11	-20
C7_MCODE_2	GO Biological Processes	Cotranslational protein targeting to membrane	11	-20
C7_MCODE_2	GO Biological Processes	Protein targeting to ER	11	-19
C7_MCODE_2	Reactome Gene Sets	SRP-dependent cotranslational protein targeting to membrane	11	-19
C7_MCODE_2	GO Biological Processes	Establishment of protein localization to endoplasmic reticulum	11	-19
C7_MCODE_3	Reactome Gene Sets	PD-1 signaling	7	-19
C7_MCODE_3	Reactome Gene Sets	Generation of second messenger molecules	7	-17
C7_MCODE_3	Reactome Gene Sets	Translocation of ZAP-70 to Immunological synapse	6	-16
C7_MCODE_3	Reactome Gene Sets	Phosphorylation of CD3 and TCR zeta chains	6	-15
C7_MCODE_3	Reactome Gene Sets	Costimulation by the CD28 family	7	-15
C7_MCODE_4	KEGG Pathway	Chemokine signaling pathway	5	-9.3
C7_MCODE_4	Reactome Gene Sets	Chemokine receptors bind chemokines	4	-8.5
C7_MCODE_4	GO Biological Processes	Chemokine-mediated signaling pathway	4	-7.8
C7_MCODE_4	GO Biological Processes	Response to chemokine	4	-7.6
C7_MCODE_4	GO Biological Processes	Cellular response to chemokine	4	-7.6
C7_MCODE_5	GO Biological Processes	Regulation of I-kappaB kinase/NF-kappaB signaling	4	-6.8
C7_MCODE_5	Reactome Gene Sets	Regulation of TNFR1 signaling	3	-6.7
C7_MCODE_5	GO Biological Processes	I-kappaB kinase/NF-kappaB signaling	4	-6.5
C7_MCODE_5	Reactome Gene Sets	TNF signaling	3	-6.4
C7_MCODE_5	GO Biological Processes	Regulation of tumor necrosis factor-mediated signaling pathway	3	-6.1

## Discussion

In spite of the great advances in tumor immunotherapy, the heterogeneity of immune cells within tumors confounded the understanding of underlying mechanisms and the predictive performance on outcomes (17). T cells are important cellular targets in tumor immunotherapy, and TILs can be used to predict clinical responses and survival of patients (30). The scRNA-seq technologies show incomparable superiority in the assessment of tumor cellular heterogeneity, which represents an ongoing challenge in cancer treatment (16). In this study, using the scRNA-seq data of isolated T cells from the tumor tissues and peripheral blood of CRC patients which were moderately differentiated, we found that the T cells in the peripheral blood (CD4^+^/CD8^+^ Naive T cells, CD4^+^/CD8^+^ central memory T cell, CD4^+^/CD8^+^ recently activated effector memory T cells and Blood-Treg) showed diverse phenotypes compared to those of the T cells in the tumor tissue (tumor-Treg, CD4^+^/CD8^+^T_RM_ T cell, CD4^+^/CD8^+^ effector memory T cells, Th17 cells, CD8^+^ exhausted T cell, and CD8^+^ intraepithelial lymphocytes), and this finding is consistent with that of Azizi et al. (17). Using unsupervised cluster analysis, we identified eight T cell types from tumor tissue, including tumor-Treg, CD4^+^/CD8^+^T_RM_ T cell, CD4^+^/CD8^+^ effector memory T cells, Th17 cells, CD8^+^ exhausted T cells, and CD8^+^ intraepithelial lymphocytes. Those T cell types may represent the major tumor-infiltrating T cell subsets in moderately differentiated CRC, and could be more specific cellular targets for the clinical immunotherapy of moderately differentiated CRC patients.

T_RM_T cells are a recently found lymphocyte lineage specialized by some memory T cells for life and function within tissues (not present within peripheral blood) (31). T_RM_ cells function in the enhancement of protective immunity, and its characteristics in tumors always are related to favorable outcomes (32). We found that CD8^+^T_EM_ showed a strong correlation with different cell clusters, including CD4^+^T_EM_, CD8^+^IEL, CD8^+^ T_RM_, and CD8^+^ T_EX_. Hence, we speculated that CD8^+^T_EM_ may be a more important tumor-infiltrating T cell type in moderately differentiated CRC. The function of CD8^+^T_EM_ and its prognostic value in CRC, especially moderately differentiated CRC, should be further investigated. T cell exhaustion represents a state of T cell function deterioration. The strong effector functions are lost and various inhibitory receptors are expressed in exhausted T cells (T_EX_) (33). Fu et al. suggested that the tumor tissue showed high percentages of T_EX_ and Treg cells compared to those in the peripheral blood (34), suggesting that tumor tissue showed relatively more immunosuppressive phenotypes. Consistently, CD8^+^T_EX_ cells were identified from the tumor tissue but not from the peripheral blood, in our study. On the contrary, tumor microenvironments consist of various cell types that communicate by ligand-receptor pairs. Targeted ligand-receptor pairs will provide promising targets in tumor immunotherapy, such as immune checkpoint inhibitors. We found that tumor-infiltrating CD8^+^T_EX_ showed more crosstalk with other cell clusters. For example, CD8^+^T_EX_ showed crosstalk with tumor-infiltrating Treg by a CCL4-CCR8 cytokine ligand-receptor pair. The scRNA-seq approach is useful for the study of the interactions across cell types in tumor microenvironments (35). In our study, a total of 7,852 ligand-receptor pairs among eight T cell types were identified from the tumor tissue, and 4,546 ligand-receptor pairs among the seven T cell clusters were identified from the peripheral blood. For example, checkpoint ligand-receptor pairs, such as tumor Treg CD80-Th17 cell CTLA4 and tumor Treg CD274-Th17 cell PDCD1, were identified in our study.

We also found some similarities and differences in the infiltrating T cells between colon cancer and rectal cancer. Tumor-infiltrating CD8^+^IEL was found in rectal cancer but not in colon cancer, while CD8^+^ T_N_ cell was found in the peripheral blood of colon cancer patients but not in that of rectal cancer patients. A larger number of tumor-infiltrating CD8^+^ Tex (88.94%) were found in colon cancer than in rectal cancer (11.06%). In the peripheral blood, 18.09% of CD4^+^ T_EMRA_/T_EFF_ cells were found in colon cancer and 81.91% in rectal cancer, while 64.06% of CD8^+^ T_EMRA_/T_EFF_ cells were found in colon cancer and 35.96% in rectal cancer. These findings suggest a heterogeneity for tumor-infiltrating T cells between colon cancer and rectal cancer. In spite of these differences, there are also similarities in the tumor-infiltrating T cells between colon cancer and rectal cancer. For example, the number of tumor-infiltrating CD8^+^ T_RM_, Th17, CD8^+^T_EM_, and CD8^+^ T_RM_ in colon cancer was not significantly different from that of rectal cancer. In peripheral blood, the number of CD4^+^ blood-T_CM_, Blood-Treg, and CD8^+^ T_CM_ cells in colon cancer had no significant difference with that of rectal cancer.

Besides, we further analyzed the differences in molecular expression and found that tumor-Treg cells showed more DEGs, while Th17 cells showed few DEGs between colon cancer and rectal cancer. For tumor-Treg, a total of 112 genes differentially expressed (43 up-regulated and 69 down-regulated) between colon cancer and rectal cancer. The PPI network that was constructed by those genes contained two significant modules. CCR6, CXCR3, CXCR6, and CCL5 were the genes in one significant module, and they were enriched in chemokine receptors bind chemokines, chemokine-mediated signaling pathway, etc. In peripheral blood, CD4^+^ T_EMRA_/T_EFF_ and CD8^+^T_CM_ showed few DEGs, while CD4^+^ T_N_ and CD8^+^ T_EMRA_/T_EFF_ showed more DEGs between colon cancer and rectal cancer. For CD8^+^ T_EMRA_/T_EFF_, a total of 359 DEGs (69 up-regulated and 259 down-regulated) between colon cancer and rectal cancer. Those genes were mainly enriched in the adaptive immune system, T cell activation, and lymphocyte activation. Furthermore, the PPI network contained five significant modules, and the genes in different modules were implicated in different functions and pathways. For example, *HLA-DRB1*, *HLA-DPB1*, *HLA-DPA1*, *HLA-DRB5*, *CD247*, *LCP2*, *PTPN11*, and *CD3E* were genes in one significant module, and were involved in PD-1 signaling, generation of second messenger molecules, costimulation by the CD28 family, etc. Naive T cells are resting cells and are important for normal T cell homeostasis (36). IELs are a larger population of lymphocytes in intestinal epithelium constituting a part of the intestinal mucosal barrier (37). Memory T cell populations include T_CM_ and T_EM_ populations, which have different homing capacity and effector function. T_EM_ cells mediate protective memory with immediate effector function, while T_CM_ cells mediate reactive memory with less or without effector function (38).

The differences in tumor-infiltrating T cells between colon cancer and rectal cancer provide insights into the study of the characteristics of tumor-infiltrating immune cells of colon and rectal cancers, and provide potential cellular targets and molecular targets in the immunotherapy of colon cancer and rectal cancer. Although only five samples were included in our study, we profiled 1632 T cells from the tumor tissue and 1252 cells from the peripheral blood using scRNA-seq. Based on 644 isolated glomerular cells, Fu et al. revealed the dynamic changes in diabetic kidney disease using scRNA-seqanalysis (39). Therefore, the sample size is enough to characterize the tumor-infiltrating T cells. In addition, only the five CRC patients with moderate differentiation were selected, which eliminated the confounding factors from different differentiation degree. Besides, multiple infiltrating T cells, including CD8^+^ T cells (CD3^+^ and CD8^+^), T helper cells (CD3^+^, CD4^+^, and CD25^−^), and regulatory T cells (CD3^+^, CD4^+^, and CD25 high) were collected, suggesting that our characterization of tumor-infiltrating T cells was relatively comprehensive. After scRNA-seq, clustering analysis was performed, which contributed to the identification of more functionally distinct T cell populations.

## Conclusions

Based on scRNA-seq analysis, we characterized the T cell subsets in CRC tumor tissue and peripheral blood. The T cells in peripheral blood showed diverse phenotypes in comparison with that of tumor tissue, and exhausted T cells were identified in tumor tissue. Various ligand-receptor interactions were identified across cell types. Colon cancer and rectal cancer also showed differences in the composition of tumor-infiltrating T cell populations. These findings can promote a better understanding of the mechanisms underlying tumor-infiltrating T cells in the progression of CRC and provide potential targets in the immunotherapy of CRC.

## Data Availability Statement

Publicly available datasets were analyzed in this study. This data can be found here: EGA (accession number EGAS00001002791); Gene Expression Omnibus (GEO) (accession number GSE108989).

## Author Contributions

All authors participated in the conception and design, data acquisition, analysis and interpretation of the study. XY, QQ, YP, and QZ wrote the manuscript. SH, JZ, and YW analyzed the data, designed, and drew. JX and MP reviewed the paper and sorted out the literature. All authors contributed to the article and approved the submitted version.

## Funding

This work was supported by the Zhejiang Provincial Natural Science Foundation (LQ20H160001), Major Science and Technology Projects for Medical and Health Care of Zhejiang Province (no. WKJ-ZJ-2013).

## Conflict of Interest

The authors declare that the research was conducted in the absence of any commercial or financial relationships that could be construed as a potential conflict of interest.
