# Supplementary material for: Single-Cell Analysis Reveals Characterization of Infiltrating T Cells in Moderately Differentiated Colorectal Cancer
Source: Front Immunol. 2021 Jan 22;11:620196. doi: 10.3389/fimmu.2020.620196 (PMC7873865; doi:10.3389/fimmu.2020.620196)
Supplement: Supplementary file 1 [file DataSheet_1.docx]

**Supplemental tables**

**Table S1** Clinical characteristics of the five colorectal cancer (CRC) patients in this study

| Patients | Age | Gender | Histological type | Stage | Grade |
| --- | --- | --- | --- | --- | --- |
| 1 | 66 | Female | Colon ADC | Ⅱ | moderate-differentiated |
| 2 | 65 | Female | Colon ADC | Ⅲb | moderate-differentiated |
| 3 | 82 | Female | Colon ADC | Ⅲb | moderate-differentiated |
| 4 | 55 | Male | Rectum ADC | Ⅲc | moderate-differentiated |
| 5 | 75 | Male | Rectum ADC | Ⅱb | moderate-differentiated |

**Table S2** Statistics of number of cells of the each known cell type in our obtained cell clusters (Analysis results for data from tumor tissue)

| **(a) CD4+ T cell clusters** | | | | | | | | | | | | | |
| --- | --- | --- | --- | --- | --- | --- | --- | --- | --- | --- | --- | --- | --- |
|  | C1 | C10 | C11 | C12 | C13 | C2 | C3 | C4 | C5 | C6 | C7 | C8 | C9 |
| CD4_C01-CCR7 | 0 | 0 | 0 | 0 | 0 | 0 | 3 | 1 | 0 | 0 | 0 | 0 | 0 |
| CD4_C02-ANXA1 | 0 | 0 | 0 | 1 | 1 | 0 | 2 | 0 | 0 | 1 | 0 | 1 | 0 |
| CD4_C03-GNLY | 0 | 1 | 0 | 0 | 0 | 0 | 0 | 0 | 0 | 0 | 0 | 0 | 0 |
| CD4_C04-TCF7 | 0 | 0 | 0 | 0 | 0 | 0 | 3 | 13 | 0 | 3 | 0 | 0 | 0 |
| CD4_C05-CXCR6 | 0 | 1 | 0 | 30 | 2 | 0 | 26 | 30 | 3 | 13 | 0 | 12 | 6 |
| CD4_C06-CXCR5 | 0 | 0 | 0 | 1 | 1 | 2 | 3 | 6 | 3 | 2 | 0 | 0 | 0 |
| CD4_C07-GZMK | 0 | 42 | 0 | 1 | 0 | 0 | 5 | 0 | 0 | 8 | 0 | 5 | 35 |
| CD4_C08-IL23R | 0 | 0 | 1 | 26 | 16 | 1 | 1 | 0 | 6 | 4 | 0 | 10 | 1 |
| CD4_C09-CXCL13 | 0 | 0 | 0 | 17 | 12 | 0 | 0 | 0 | 1 | 7 | 0 | 5 | 4 |
| CD4_C10-FOXP3 | 0 | 0 | 0 | 0 | 0 | 0 | 0 | 2 | 0 | 0 | 0 | 1 | 0 |
| CD4_C11-IL10 | 0 | 0 | 0 | 1 | 0 | 0 | 1 | 0 | 7 | 1 | 0 | 0 | 0 |
| CD4_C12-CTLA4 | 88 | 0 | 124 | 1 | 4 | 99 | 0 | 0 | 40 | 3 | 82 | 0 | 0 |
| MAIT.CD4 | 0 | 0 | 0 | 0 | 1 | 0 | 0 | 0 | 0 | 0 | 0 | 0 | 0 |
| diverse.DN | 2 | 15 | 6 | 15 | 2 | 32 | 14 | 31 | 15 | 34 | 5 | 5 | 34 |
| diverse.DP | 0 | 0 | 0 | 1 | 0 | 0 | 0 | 0 | 0 | 0 | 0 | 0 | 0 |
| diverse.other | 1 | 7 | 6 | 6 | 3 | 10 | 2 | 4 | 9 | 3 | 4 | 6 | 9 |
| iNKT.DN | 0 | 0 | 0 | 0 | 0 | 0 | 0 | 1 | 0 | 0 | 0 | 0 | 0 |
| **(b) CD8+ T cell clusters** | | | | | | | | | | | | | |
|  | C1 | C10 | C11 | C12 | C2 | C3 | C4 | C5 | C6 | C7 | C8 | C9 |  |
| CD8_C01-LEF1 | 0 | 0 | 0 | 0 | 0 | 3 | 0 | 0 | 0 | 0 | 0 | 0 |  |
| CD8_C02-GPR183 | 0 | 0 | 0 | 0 | 0 | 6 | 0 | 0 | 0 | 0 | 0 | 1 |  |
| CD8_C03-CX3CR1 | 0 | 0 | 0 | 0 | 0 | 0 | 0 | 0 | 5 | 0 | 0 | 1 |  |
| CD8_C04-GZMK | 6 | 3 | 47 | 3 | 0 | 15 | 3 | 16 | 27 | 2 | 4 | 41 |  |
| CD8_C05-CD6 | 17 | 0 | 1 | 0 | 0 | 5 | 7 | 1 | 0 | 0 | 9 | 1 |  |
| CD8_C06-CD160 | 2 | 0 | 1 | 0 | 0 | 0 | 28 | 0 | 0 | 0 | 4 | 0 |  |
| CD8_C07-LAYN | 0 | 43 | 3 | 53 | 40 | 0 | 0 | 5 | 10 | 42 | 1 | 0 |  |
| CD8_C08-SLC4A10 | 3 | 0 | 1 | 0 | 1 | 1 | 0 | 0 | 0 | 0 | 0 | 0 |  |
| MAIT.other | 2 | 0 | 0 | 0 | 0 | 0 | 0 | 0 | 0 | 0 | 0 | 0 |  |
| diverse.DN | 1 | 0 | 0 | 0 | 0 | 0 | 5 | 3 | 0 | 1 | 0 | 1 |  |
| diverse.DP | 0 | 0 | 0 | 0 | 0 | 0 | 1 | 0 | 0 | 0 | 0 | 0 |  |
| diverse.other | 5 | 1 | 4 | 1 | 2 | 2 | 10 | 4 | 0 | 7 | 1 | 3 |  |

**Table S3** Statistics of number of cells of the each known cell type in our obtained cell clusters (Analysis results for data from peripheral blood)

| **(a) CD4+ T cell clusters** | | | | | | | | | | | | | |
| --- | --- | --- | --- | --- | --- | --- | --- | --- | --- | --- | --- | --- | --- |
|  | C1 | C10 | C11 | C12 | C13 | C2 | C3 | C4 | C5 | C6 | C7 | C8 | C9 |
| CD4_C01-CCR7 | 4 | 0 | 70 | 0 | 1 | 0 | 0 | 54 | 23 | 25 | 8 | 2 | 0 |
| CD4_C02-ANXA1 | 27 | 30 | 11 | 4 | 5 | 1 | 1 | 1 | 0 | 1 | 35 | 5 | 2 |
| CD4_C03-GNLY | 0 | 2 | 0 | 1 | 0 | 23 | 40 | 0 | 0 | 0 | 0 | 4 | 0 |
| CD4_C05-CXCR6 | 0 | 0 | 0 | 0 | 0 | 0 | 1 | 0 | 0 | 0 | 0 | 0 | 0 |
| CD4_C10-FOXP3 | 4 | 2 | 1 | 15 | 26 | 0 | 0 | 0 | 0 | 15 | 14 | 3 | 40 |
| CD4_C12-CTLA4 | 0 | 0 | 0 | 1 | 0 | 0 | 0 | 0 | 0 | 0 | 0 | 0 | 1 |
| MAIT.CD4 | 0 | 0 | 0 | 0 | 1 | 0 | 0 | 0 | 0 | 0 | 0 | 0 | 0 |
| diverse.DN | 22 | 12 | 17 | 4 | 18 | 13 | 3 | 38 | 28 | 34 | 12 | 12 | 7 |
| diverse.other | 5 | 6 | 6 | 4 | 3 | 4 | 8 | 8 | 6 | 6 | 8 | 1 | 9 |
| iNKT.CD4 | 1 | 0 | 0 | 0 | 0 | 0 | 0 | 0 | 0 | 0 | 0 | 0 | 0 |
| **(b) CD8+ T cell clusters** | | | | | | | | | | | | | |
|  | C1 | C2 | C3 | C4 | C5 | C6 | C7 |  |  |  |  |  |  |
| CD8_C01-LEF1 | 9 | 0 | 0 | 20 | 0 | 1 | 0 |  |  |  |  |  |  |
| CD8_C02-GPR183 | 30 | 4 | 0 | 9 | 0 | 0 | 10 |  |  |  |  |  |  |
| CD8_C03-CX3CR1 | 1 | 63 | 127 | 0 | 51 | 45 | 25 |  |  |  |  |  |  |
| CD8_C04-GZMK | 0 | 1 | 0 | 0 | 6 | 0 | 4 |  |  |  |  |  |  |
| CD8_C05-CD6 | 0 | 0 | 0 | 0 | 0 | 0 | 1 |  |  |  |  |  |  |
| CD8_C06-CD160 | 0 | 0 | 0 | 0 | 0 | 0 | 1 |  |  |  |  |  |  |
| CD8_C08-SLC4A10 | 0 | 7 | 0 | 0 | 0 | 1 | 2 |  |  |  |  |  |  |
| MAIT.DN | 0 | 1 | 0 | 0 | 0 | 1 | 0 |  |  |  |  |  |  |
| MAIT.other | 0 | 1 | 0 | 0 | 0 | 1 | 0 |  |  |  |  |  |  |
| diverse.DN | 0 | 2 | 0 | 0 | 0 | 3 | 0 |  |  |  |  |  |  |
| diverse.DP | 0 | 1 | 0 | 0 | 0 | 0 | 0 |  |  |  |  |  |  |
| diverse.other | 3 | 5 | 8 | 0 | 2 | 4 | 3 |  |  |  |  |  |  |
