# Supplementary figures and images for: Single-Cell Analysis Reveals Characterization of Infiltrating T Cells in Moderately Differentiated Colorectal Cancer
Source: Front Immunol. 2021 Jan 22;11:620196. doi: 10.3389/fimmu.2020.620196 (PMC7873865; doi:10.3389/fimmu.2020.620196)

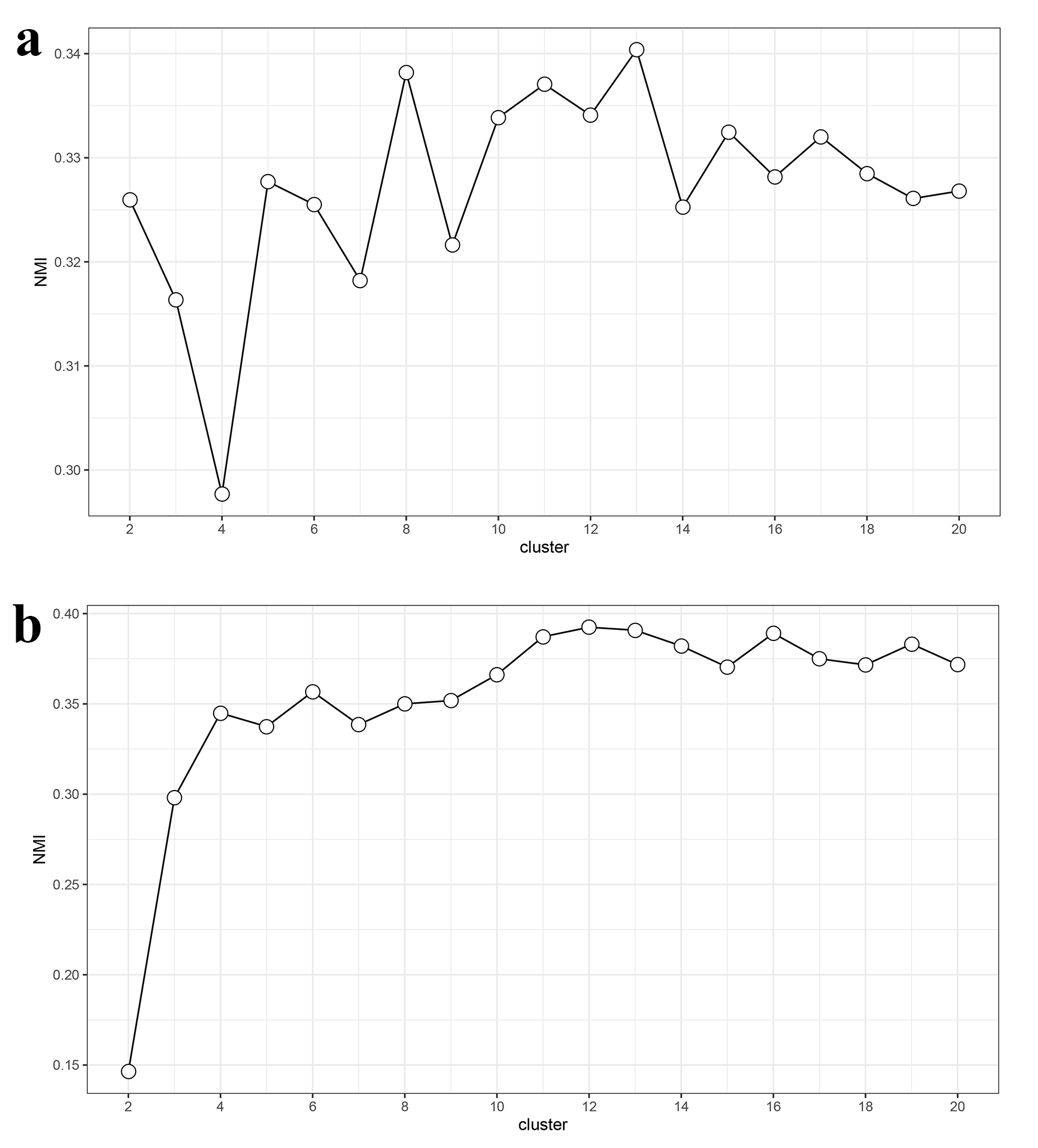

Supplement: Supplementary file 2 [file Image_1.tif]

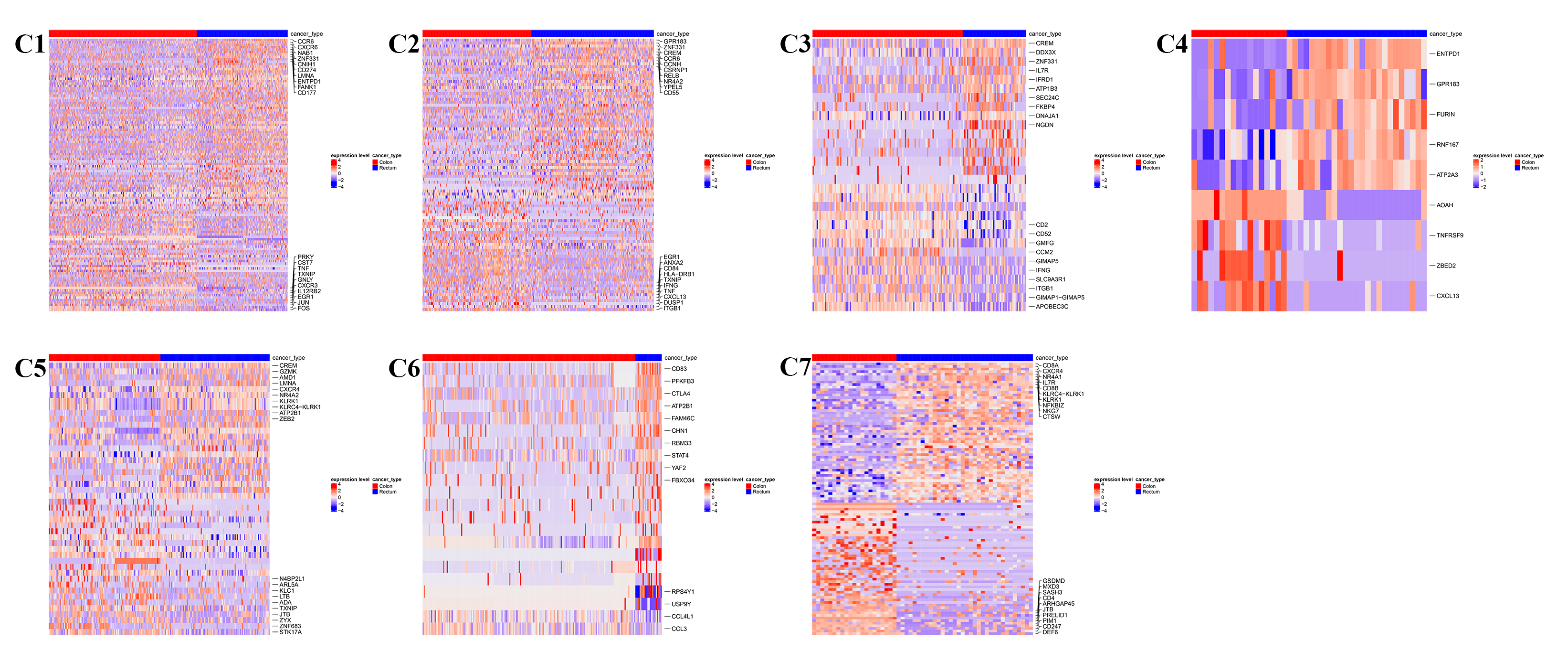

Supplement: Supplementary file 3 [file Image_2.tif]

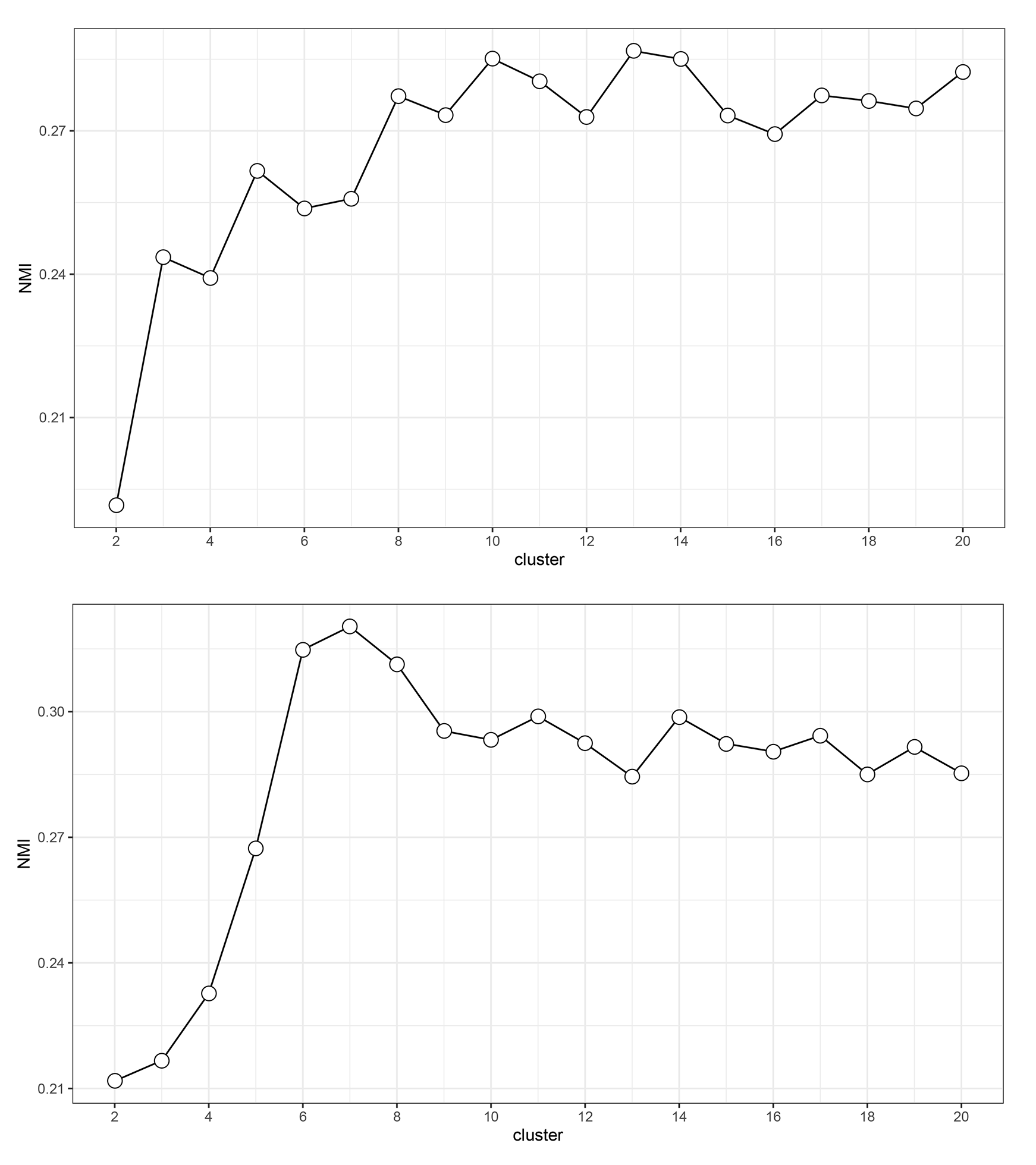

Supplement: Supplementary file 4 [file Image_3.tif]

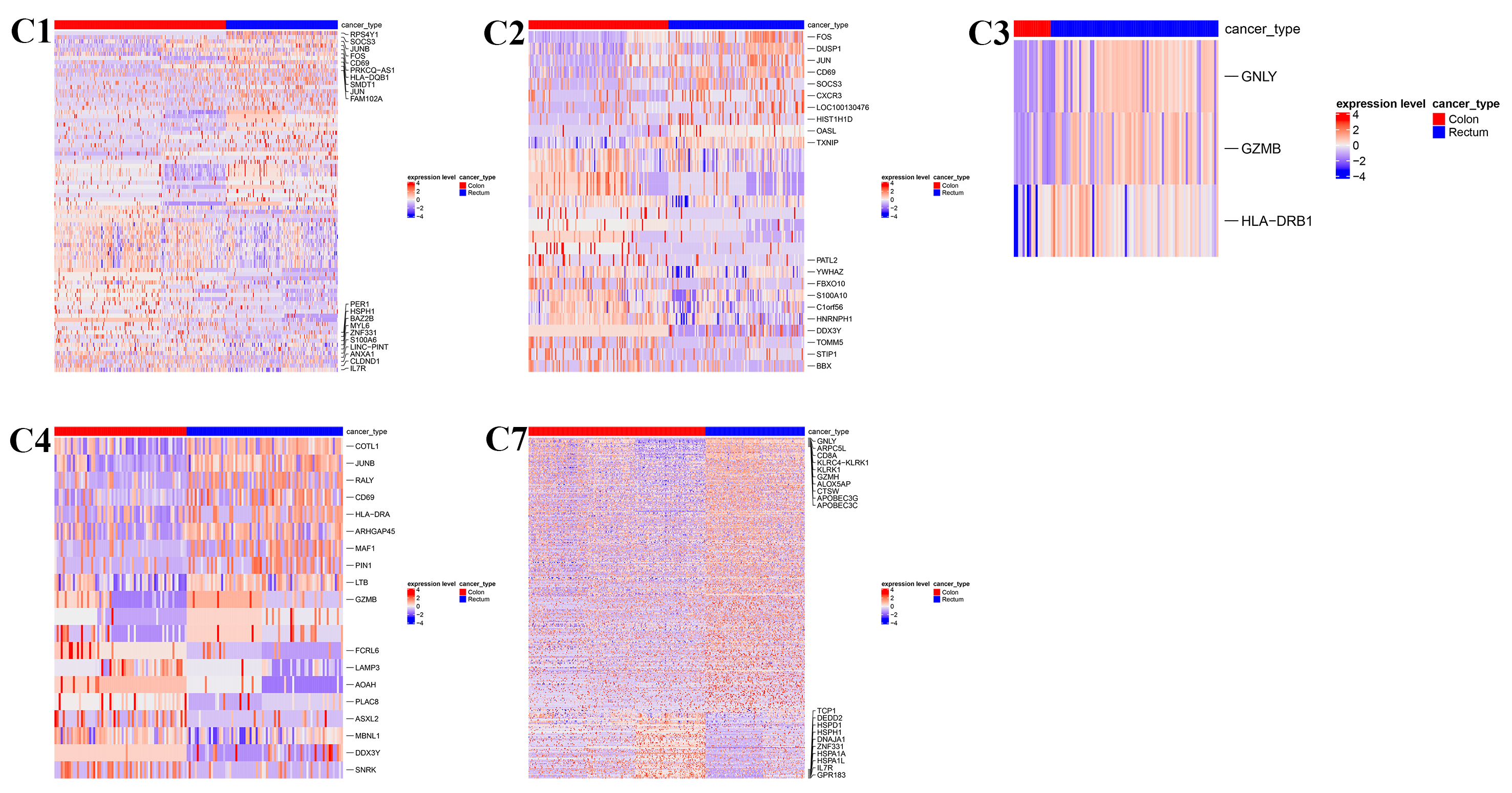

Supplement: Supplementary file 5 [file Image_4.tif]
